# AGING IN PLACE: EFFECTIVE INTERPROFESSIONAL COLLABORATION AND ENGAGEMENT WITH OLDER ADULTS

**DOI:** 10.1093/geroni/igz038.487

**Published:** 2019-11-08

**Authors:** Elizabeth Fine-Smilovich, Diana L Morris, David M Rosenberg, Elizabeth O’Toole, Cynthia Booth-Lord, Klara K Papp, Patricia A Thomas

**Affiliations:** 1 Case Western Reserve University School of Medicine, Cleveland, Ohio, United States; 2 Case Western Reserve University, Cleveland, Ohio, United States; 3 Case Western University School of Medicine, Cleveland, Ohio, United States; 4 MetroHealth/Case Western Reserve University School of Medicine, Cleveland, United States

## Abstract

Background: An innovative educational program addresses two gaps in health professions education: lack of an emerging workforce comfortable caring for older adults and proficiency in working in an interprofessional (IP) setting. We sought to explore whether AIP provides grounding in pillars of IPE and geriatric competencies through experiential learning in IP teams with older adults in a community setting. Methods: Early health profession students n=37 (MD, MSN, PA, SW), working in teams of 3, made monthly visits to older adults’ residences over a one-year period. Workshops on core geriatric and IPE principles defined expected learning goals for client visits. Visits were followed by: 1) written field notes; 2) reflections based on pre-determined learning prompts; and 3) debriefing sessions with faculty members. Students completed pre and post program questionnaires including Attitudes Towards Social Issues in Medicine, Geriatrics Attitude Scale, ICCAS, and RIPLS. Pre-post results were analyzed using t-tests and qualitative analysis of comments. Results: 25 (68%) students completed pre-post questionnaires. Responses on the interprofessional collaboration scale significantly increased following the program (t=2.09; p = 0.047) and 94% responded that they could “well” or “very well” describe issues that impact older adults’ health, quality of life, and convey appreciation toward older adults. Discussion: Students, engaging with older adults longitudinally in a community setting learned pillars of IPE, geriatric care competencies, and gained insights into this population. An interprofessional, experiential learning program is feasible and effective way to increase interest and self-efficacy in working with older adult populations.

